# Acarbose Accelerates Wound Healing via Akt/eNOS Signaling in* db/db* Mice

**DOI:** 10.1155/2017/7809581

**Published:** 2017-03-08

**Authors:** Xue Han, Yaping Deng, Jiawen Yu, Yuannan Sun, Guofei Ren, Jian Cai, Jianjun Zhu, Guojun Jiang

**Affiliations:** Department of Pharmacy, Xiaoshan Hospital, Hangzhou, Zhejiang 311202, China

## Abstract

Refractory wound is a dreaded complication of diabetes and is highly correlated with EPC dysfunction caused by hyperglycemia. Acarbose is a widely used oral glucose-lowering drug exclusively for T2DM. Previous studies have suggested the beneficial effect of acarbose on improving endothelial dysfunction in patients with T2DM. However, no data have been reported on the beneficial efficacy of acarbose in wound healing impairment caused by diabetes. We herein investigated whether acarbose could improve wound healing in T2DM* db/db* mice and the possible mechanisms involved. Acarbose hastened wound healing and enhanced angiogenesis, accompanied by increased circulating EPC number in* db/db* mice. In vitro, a reversed BM-EPC dysfunction was observed after the administration of acarbose in* db/db* mice, as reflected by tube formation assay. In addition, a significantly increased NO production was also witnessed in BM-EPCs from acarbose treated* db/db* mice, with decreased O_2_ levels. Akt inhibitor could abolish the beneficial effect of acarbose on high glucose induced EPC dysfunction in vitro, accompanied by reduced eNOS activation. Acarbose displayed potential effect in promoting wound healing and improving angiogenesis in T2DM mice, which was possibly related to the Akt/eNOS signaling pathway.

## 1. Introduction

Diabetes mellitus (DM), characterized by hyperglycemia, can cause many sever health complications including cardiovascular diseases, kidney failure, and lower-extremity amputations [1]. Wound healing, in particular, is greatly influenced by diabetes [2, 3] and has been extensively studied. It has been demonstrated that individuals with diabetes exhibit reduced capability in wound healing and are more vulnerable to developing serious chronic foot ulcers, which extremely affects the quality of patients' life [4, 5]. Therefore, it is imperative to explore effective therapies and elucidate the underlying mechanisms for the conquering of diabetes-induced impaired wound healing.

It is widely accepted that endothelial dysfunction is vital in vascular diseases and is the primary factor of impaired wound healing [6, 7]. Endothelial precursor cells (EPCs), immature endothelial cells, have attracted enormous attention due to their ability of differentiating into mature endothelial cells [8, 9], which in turn contributes to endothelial regeneration and neovascularization [10]. Clinical studies have observed both reduction in the amount of circulating EPCs and dysfunction of these cells in diabetic patients [11, 12]. Therefore, EPC dysfunction and the consequent abnormality of endothelial regeneration may influence the susceptibility to developing impaired wounding healing under diabetes.

Acarbose, an *α*-glucosidase inhibitor (AGI), is a commonly used oral glucose-lowering drug for the treatment of type 2 diabetes mellitus (T2DM) [13, 14] and could inhibit the conversion of carbohydrates into monosaccharides thus suppressing the intestinal absorption so that the bioavailability of carbohydrates is declined in the body and the blood glucose levels are significantly lowered [15]. The postprandial surge of plasma glucose could lead to severe endothelial dysfunction in diabetic patients [16–18]. Clinical researches have shown that the administration of acarbose improved endothelial function and reduced the risk of cardiovascular events in patients with T2DM [19–21]. However, up to now, there is limited information about the link between acarbose and wound healing, and the direct effects of acarbose on high glucose impaired EPC function.

On the basis of these findings, we hypothesize that acarbose may accelerate diabetes-induced wound healing via improving EPC function. To test this hypothesis, this study sought to determine the effect of acarbose on wound healing and EPCs in* db/db* diabetic mice.

## 2. Materials and Methods

### 2.1. Animals

Male C57BL/KsJ mice and BKS.Cg-m +/+ Lepr^db^/J* db*/*db* mice (*db*/*db*) with a C57BL/KsJ background were purchased from the Sino-British SIPPR/BK Lab Animal Ltd. (Shanghai, China). Mice were maintained in microisolator cages on a 12 h light/dark cycle with controlled temperature (23 ± 2°C) and humidity (about 70%) and received a standard laboratory pellet diet and water ad libitum. All animals were cared for in accordance with institutional animal care guidelines and the Guide for Care and Use of Laboratory Animals published by the National Institutes of Health. Detailed genotyping methods for identification of the mouse leptin receptor mutation (C57BL/KsJ-db/db) are described in the Data Supplement in Supplementary Material available online at https://doi.org/10.1155/2017/7809581.

### 2.2. Reagents and Antibodies

Acarbose was obtained from Sigma-Aldrich. The Akt inhibitor MK-2206 2HCl was obtained from Selleck (Shanghai, China). Rabbit anti-Akt (Cat. No. 4691), anti-phosphorylated Akt (Ser473, Cat. No. 4060), anti-endothelial nitric oxide synthase (eNOS, Cat. No. 9572), and anti-phosphorylated-eNOS (Ser1177, Cat. No. 9571) monoclonal antibodies were purchased from Cell Signaling Technology (MA, USA). Mouse anti-GAPDH (Cat. No.AG019) monoclonal antibody was purchased from Beyotime (Shanghai, China). Secondary antibodies including goat anti-rabbit antibody IgG-HRP and goat anti-mouse IgG-HRP were purchased from EarthOx (USA).

### 2.3. Experimental Protocols

**Figure 1:**
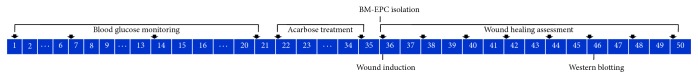
Experimental schedule. The blood glucose of* db/db* diabetic mice was monitored every 7 day until day 21; then acarbose (50 mg/kg/d,* i.g.*) treatment was conducted for consecutive 14 days. At last, wound healing models were created and BM-EPCs were collected.

C57BL/6J mice were used as controls. Blood glucose was monitored at the indicated time using a blood glucose monitoring system (Maochang, Taipei, China) with whole blood obtained from the tail veins of the mice. On day 22, the mice with blood glucose levels greater than 250 mg/dL were defined as* db/db* diabetic mice and subsequently treated with acarbose (50 mg/kg/d,* i.g.*) treatment or vehicle (0.5% CMC-Na) for consecutive 14 days. The control mice received vehicle. On day 36, the mice were used for wound healing experiments or anesthetized for harvesting the bone marrow to isolate the BM-EPCs (Figure 1).

### 2.4. Analysis of Wound Healing and Angiogenesis

After being anesthetized with ketamine (100 mg/kg,* i.p.*), mice were fixed, dehaired on the dorsum, and swabbed with betadine and 75% ethanol three times [22]. A 6 mm circular wound was produced by punch biopsy, and the closure of the wounded area was measured every 2 days until day 14 using a clear, bioclusive transparent dressing (Johnson & Johnson, Arlington, TX, USA). The photographed wound area was calculated with Image-Pro Plus software (Media Cybernetics, Silver Spring, MD, USA).

The assessment of angiogenesis and SDF-1*α* detection were performed using CD31 and SDF-1*α* immunochemistry and hematoxylin (VWR Scientific, Radnor, PA, USA) staining [23, 24]. Briefly, punch biopsy of the skin at the wounded area was conducted on days 7 and 14. The fixed skin samples were embedded in paraffin followed by deparaffinization and rehydration. After being immersed in Tris-buffered saline (pH 7.5) for 5 min, the slides were subjected to blocking of endogenous peroxidase. After blocking with serum for 30 min (Vector Laboratories, Burlingame, CA, USA), the slides were incubated with an anti-CD31 antibody (10 *μ*g/ml, 1 : 50; BD Bioscience, San Jose, CA, USA) and SDF-1*α* (1 : 50; Abcam, Cambridge, MA, USA) for 60 min at room temperature followed by additional incubation with a biotinylated secondary antibody (anti-mouse IgG, Vectastain Elite ABC kit, Vector Laboratories) for 30 min, Vectastain Elite ABC Reagent (Vector Laboratories, Burlingame, CA, USA) for 30 min, and Nova Red (Vector Laboratories, Burlingame, CA, USA) for 15 min. Then, the slides were counterstained with hematoxylin for 10 sec before differentiation in 1% aqueous glacial acetic acid and rinsing in running tap water. The capillaries were depicted as CD31-positive tubular structures, and capillary density in the wounded area was quantified. One slide from each mouse was examined, and for each slide, two high-power fields (200x) were determined. The data were summed and averaged as the capillaries per high-power field.

### 2.5. Circulating EPCs Measurement

Blood samples of approximately 0.5 ml were collected from the anesthetized mice and then placed in heparin pretreated tubes [25]. After being mixed with PBS (1 : 1), 1 ml of gradient centrifugation liquid 1083 (Sigma, St. Louis, MO, USA) was added to the diluted blood samples followed by centrifugation (3,000 rpm) for 25 min at room temperature. The mononuclear cell fraction was transferred to a new tube containing erythrocytes lysis buffer solution for 5 min. After centrifugation and wash, cells were suspended in buffer solutions containing FITC-Sca-1 (eBioscience, San Diego, CA, USA) and PE-Flk-1 (BD, San Jose, CA, USA) antibodies for an incubation of 1 h at room temperature. The circulating EPCs of Sca^+^Flk-1^+^ cells were detected using flow cytometry analysis.

### 2.6. BM-EPC Extraction

Mouse bone marrow-derived EPCs (BM-EPCs) were extracted and cultured according to the previous method [26].

*Tube Formation Assay*. Seven days after BM-EPC culture, cells were trypsinized and collected. A total of 3 × 10^4^ cells were planted into 96-well plates precoated with growth factor-induced Matrigel (BD Biosciences, Bedford, MA, USA) followed by incubation for 6 h at 37°C. At the end of time, the tubes were observed under inverted phase-contrast microscope (Leica Microsystems Inc., Wetzlar, Germany). Images of the tubes were obtained from five randomly selected microscopic fields (50x) per sample, and neovascularization of BM-EPCs was determined.

*Migration Assay*. A total of 5 × 10^4^ EPCs was plated in the upper chambers of 24-well Transwell plates (Corning Transwell, Lowell, MA) per well. Medium containing VEGF (50 ng/ml) was placed in the lower chambers followed by 24 h of incubation at 37°C. Then, cells were fixed with 2% paraformaldehyde and stained by Hoechst33258 (Sigma-Aldrich, St. Louis, MO). The stained cells were observed under the fluorescence microscope.

*Adhesion Assay*. The cells with a concentration of 5 × 10^5^/ml were plated in 96-well plates coated with mouse vitronectin 1 *μ*g/ml. After incubation for 2 h, nonadherent cells were washed by PBS and adherent cells were fixed with 2% paraformaldehyde. The cells stained by Hoechst33258 and were counted in 5 random low-power (50x) microscopic fields per sample.

### 2.7. Analysis of BM-EPC Function

Tube formation, migration, and adhesion assays of BM-EPCs were used for evaluation of BM-EPC angiogenic capacity [26, 27]. Briefly, 7 days after BM-EPC culture, cells were trypsinized and collected. A total of 3 × 10^4^ cells were planted into 96-well plates precoated with growth factor-induced Matrigel (BD Biosciences, Bedford, MA, USA) followed by incubation for 6 h at 37°C. At the end of time, the tubes were observed under inverted phase-contrast microscope (Leica Microsystems Inc., Wetzlar, Germany). Images of the tubes were obtained from five randomly selected microscopic fields (50x) per sample, and neovascularization of BM-EPCs was determined.

### 2.8. Measurement of Intracellular NO and O_2_^−^

Intracellular NO level was detected as previously described [26]. Briefly, after 7 days of cultivation, BM-EPCs were collected and incubated with 10^−6^ M DAF-FM diacetate (Invitrogen, Carlsbad, CA, USA) for 30 min at 37°C and an additional staining of 30 min at room temperature in dark. After incubation, the DAF-FM fluorescence intensity was also determined by flow cytometry analysis.

For intracellular O_2_^−^ detection, cells were collected and incubated with 0.5 × 10^−6 ^M dihydroethidium (DHE, Invitrogen, Carlsbad, CA, USA) for 30 min at room temperature in dark. After incubation, the DHE fluorescence intensity was determined by flow cytometry analysis [26].

### 2.9. Western Blot Analysis

Protein was extracted and determined in accordance with previously technique [28, 29]. In brief, protein samples were obtained from BM-EPCs and subjected to quantification using BCA assay (Thermo Scientific, Rockford, IL, USA). A total of 30 *μ*g samples were run on 10% SDS-PAGE and electrotransferred to nitrocellulose membranes. The membrane was then incubated in 5% BSA/PBST for 1 h at 37°C to block unspecific binding. After rinsing, the membrane was incubated with primary antibodies for Akt, p-Akt, eNOS, or p-eNOS (Cell Signaling Technology, Beverly, MA, USA) followed by staining with IRDye 800CW-conjugated goat anti-rabbit secondary antibody (1 : 5,000; Li-Cor Bioscience, Lincoln, NE, USA). Infrared fluorescence images of specific protein bands were observed using an Odyssey infrared imaging system (Li-Cor Bioscience, Lincoln, NE, USA) and quantified with Quantity One software (Bio-Rad, Hercules, CA, USA).

### 2.10. In Vitro Assay

BM-EPCs were obtained from* db/db* and C57BL/6 mice and cultured in vitro. Seven days later, the culture medium was changed with freshly prepared high glucose (33 mM) medium or high glucose medium containing acarbose (1 *μ*M) for 24 h, acarbose (1 *μ*M) together with MK-2206 (an Akt inhibitor; 1 *μ*M), or high glucose medium containing both acarbose (1 *μ*M) and MK-2206 (1 *μ*M). Effects of acarbose on high glucose-induced EPC dysfunction were evaluated by functional analysis (tube formation) and by detecting intracellular NO and O_2_^−^ changes. Activation of Akt and eNOS was determined by western blot analysis.

### 2.11. Statistical Analysis

All data are expressed as the means ± standard deviation. Statistical significance was analyzed by one-way ANOVA followed by Newman-Keuls multiple comparison tests using GraphPad Prism Software version 5. *P* values less than 0.05 were considered statistically significant.

## 3. Results

### 3.1. Effect of Acarbose on Blood Glucose and Body Weight in* db/db* Mice

**Figure 2:**
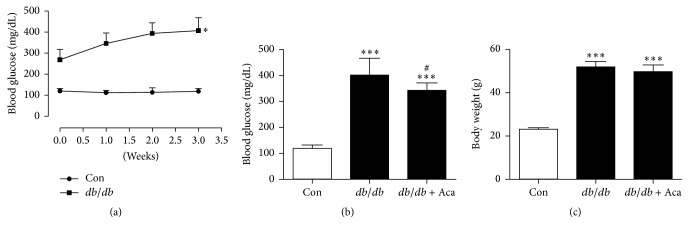
Blood glucose and body weight change in* db/db* mice. (a) Blood glucose was significantly increased in* db/db* mice compared to control. ^*∗*^*P* < 0.05 versus control. In* db/db* mice, acarbose treatment (50 mg/kg/d × 14 d,* i.g.*) significantly decreased blood glucose (b) but did not modify body weight (c). ^*∗∗∗*^*P* < 0.001 versus Con; ^#^*P* < 0.05* versus db/db*. Values are expressed as the mean ± standard deviation (*n* = 7 per group). Con, control; Aca, acarbose.

Blood glucose was significantly increased in* db/db* mice compared with the control (*P* < 0.05; Figure 2(a)). After administration of acarbose, compared to* db/db* mice, blood glucose levels were slightly but significantly reduced (342 ± 29 versus 401 ± 65 mg/dL, *P* < 0.05; Figure 2(b)). There was no significant difference in body weight between* db/db* mice with and without acarbose treatment (Figure 2(c)).

### 3.2. Acarbose Accelerated Wound Healing and Increased Angiogenesis in* db/db* Mice

**Figure 3:**
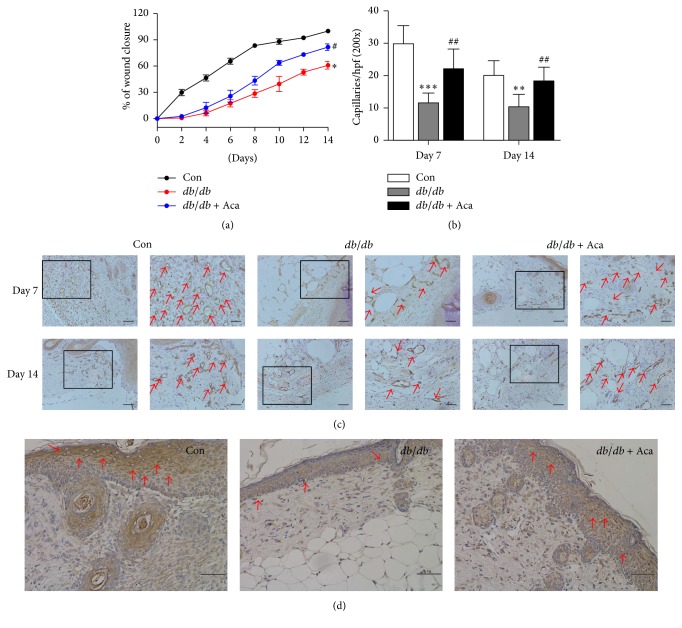
Acarbose therapy accelerated wound closure and enhanced angiogenesis in* db/db* mice. An approximate 6 mm diameter circle wound was made by punch biopsy on dorsal and wound healing was assessed every 2 days until day 14. (a) Acarbose treatment obviously accelerated wound closure in* db/db* mice compared to untreated diabetic ones. (b) Acarbose treatment significantly increased wound capillaries compared with the untreated* db/db* mice on days 7 and 14. (c) Typical photographs of CD31 staining on days 7 and 14; red arrows point to CD31-positive capillaries; boxed regions (100x; scale bar = 100 *μ*m) are shown at higher magnification (200x; scale bar = 50 *μ*m) to the right. ^*∗∗∗*^*P* < 0.001, ^*∗∗*^*P* < 0.01, and ^*∗*^*P* < 0.05 versus Con; ^##^*P* < 0.01, ^#^*P* < 0.05 versus* db/db*. (d) SDF-1*α* expression in wound site was present predominantly in acarbose-treated group on day 7; red arrows show positive brown staining for SDF-1*α*; scale bar = 50 *μ*m. Values are expressed as the mean ± standard deviation (*n* = 5 per group). Con, control; Aca, acarbose.

The wound healing abilities were significantly impaired in diabetic* db/db* mice compared with control (*P* < 0.05; Figure 3(a)). However, the process of wound healing was significantly accelerated in the acarbose-treated mice compared with* db/db* animals (*P* < 0.05; Figure 3(a)). Furthermore, angiogenesis around the wound was assessed at days 7 and 14 after wound creation. It was found that capillary density was significantly lower in the diabetic* db/db* mice compared with control. On days 7 and 14 after acarbose treatment, compared with* db/db* mice, capillary density was significantly increased (*P* < 0.01; Figures 3(b) and 3(c)). In addition, SDF-1*α* immunohistochemistry showed an increased staining in acarbose-treated wound site compared to* db/db* mice (Figure 3(d)). These results suggest that acarbose administration was able to improve wound healing impairment and increase angiogenesis in diabetic mice.

### 3.3. Acarbose Increased Circulating EPC Number and Improved EPC Function in* db/db* Mice

**Figure 4:**
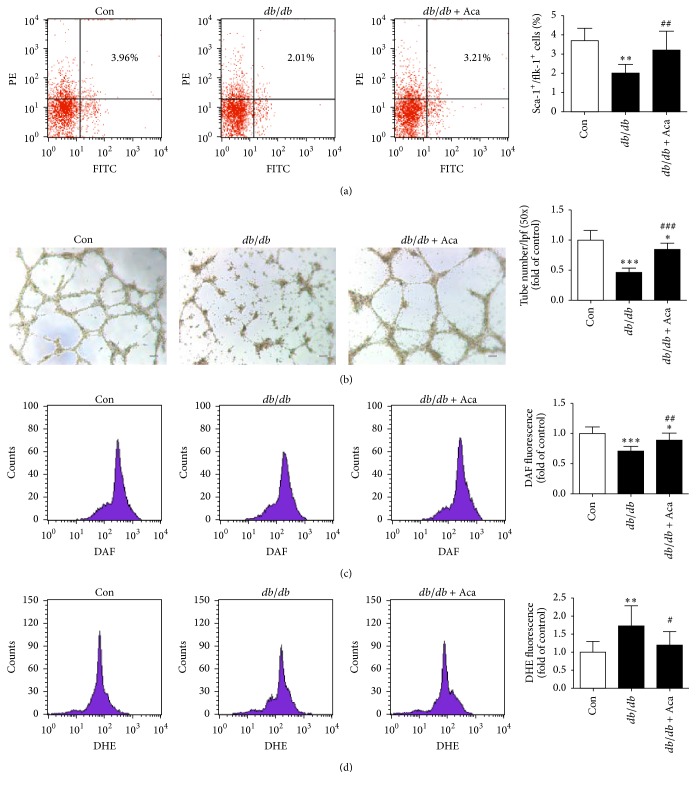
Acarbose therapy improved BM-EPC function and decreased intracellular reactive oxygen species (ROS) levels in* db/db* mice. (a) Circulating EPC numbers were detected by flow cytometry and the percentage of Sca-1^+^/Flk-1^+^ cells was calculated. Acarbose significantly increased circulating EPC number in* db/db* mice. (b) Typical images of tube formation assay of BM-EPCs. The number of tubes in each sample was calculated from 5 low-power fields (50x; scale bar = 100 *μ*m) at random. Acarbose enhanced the capacity of tube formation of BM-EPCs. (c) Intracellular NO level was determined by flow cytometry and the percentage of DAF fluorescence intensity was calculated. Acarbose obviously enhanced NO level in BM-EPCs. (d) DHE fluorescence intensity was determined by flow cytometry. Acarbose suppressed intracellular O_2_^−^ level in BM-EPCs. ^*∗∗∗*^*P* < 0.001, ^*∗∗*^*P* < 0.01, and ^*∗*^*P* < 0.05 versus Con; ^###^*P* < 0.001, ^##^*P* < 0.01, and ^#^*P* < 0.05 versus* db/db*. Values are expressed as the mean ± standard deviation (*n* = 7–9 per group). Con, control; Aca, acarbose.

The number of circulating EPCs and the function of BM-EPCs were significantly reduced in* db/db* mice when compared with the control. Acarbose treatment increased the circulating EPC number (3.21 ± 0.99 versus 2.01 ± 0.45%, *P* < 0.01; Figure 4(a)) and improved the impaired EPC function (tube formation capacity: 0.85 ± 0.10 versus 0.46 ± 0.07, *P* < 0.001; Figure 4(b)) in* db/db* mice when compared with the untreated* db/db* ones. In addition, the intracellular NO level was lower and the O_2_^−^ production was greater in* db/db* mice when compared to the control. Acarbose treatment significantly rectified these changes in* db/db* mice (NO: 0.89 ± 0.12 versus 0.71 ± 0.08, *P* < 0.01, Figure 4(c); O_2_^−^: 1.20 ± 0.37 versus 1.73 ± 0.56, *P* < 0.05, Figure 4(d)).

### 3.4. Acarbose Increased Both Phosphorylated-eNOS and Phosphorylated-Akt Expression in EPCs from* db/db* Mice

**Figure 5:**
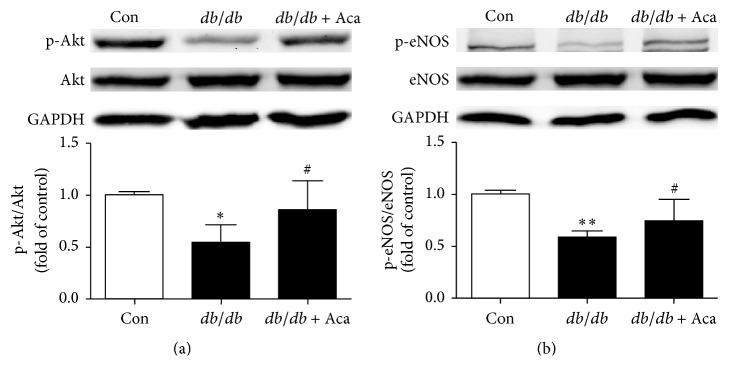
Acarbose stimulated the expression levels of activated Akt/eNOS in BM-EPCs from* db/db* mice. BM-EPCs were isolated and cultured from anesthetized mice. Akt and eNOS in BM-EPCs were conducted by western blotting, and acarbose greatly enhanced activated Akt and eNOS expression in BM-EPCs from* db/db* mice. ^*∗∗*^*P* < 0.01, ^*∗*^*P* < 0.05 versus Con; ^#^*P* < 0.05 versus* db/db*. Values are expressed as the mean ± standard deviation (*n* = 4 per group). Con, control; Aca, acarbose.

Western blot was conducted to determine the p-Akt, Akt, p-eNOS, and eNOS expression in EPCs in diabetic mice. Compared with control, it was found that both the ratio of p-Akt/Akt and p-eNOS/eNOS were significantly reduced in EPCs in* db/db* mice. Acarbose administration led to a significant increase in Akt (0.85 ± 0.28 versus 0.54 ± 0.17, *P* < 0.05; Figure 5(a)) and eNOS (0.74 ± 0.21 versus 0.59 ± 0.04, *P* < 0.05; Figure 5(b)) activation in EPCs in* db/db* mice when compared with the untreated* db/db* mice.

### 3.5. Acarbose Alleviated Dysfunction of BM-EPCs Induced by High Glucose

**Figure 6:**
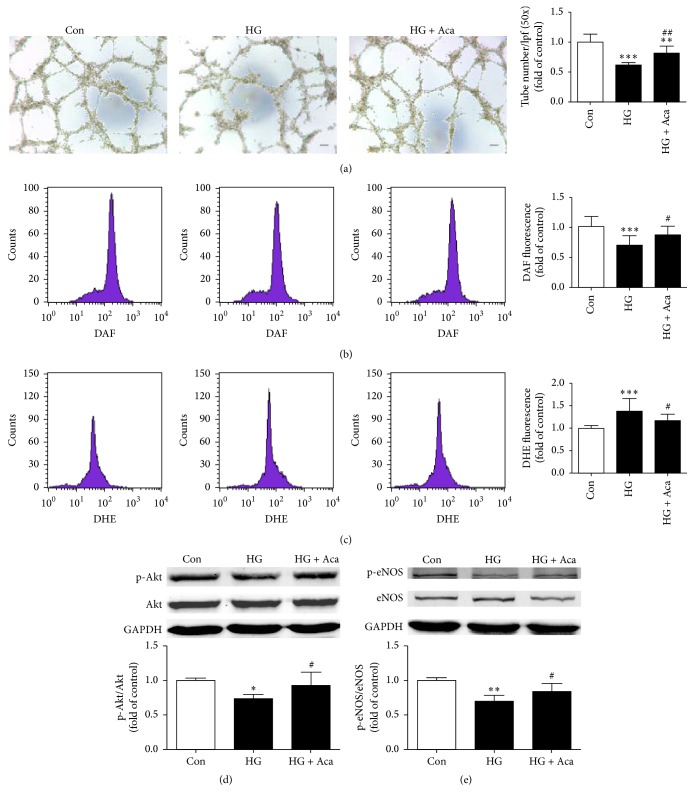
Acarbose alleviated EPC dysfunction, decreased ROS expression, and increased Akt/eNOS phosphorylated-to-total ratio in BM-EPCs induced by high glucose. BM-EPCs were isolated from normal mice and cultured under high glucose (33 mM) together with acarbose (1 *μ*M) for 24 h. (a) Assessment of tube formation ability. (b) Intracellular NO level was determined by flow cytometry and the percentage of DAF fluorescence intensity was calculated. (c) Intracellular DHE fluorescence intensity of EPCs was measured by flow cytometry. Western blot analysis was subjected to detected expression levels of activation Akt (d) and eNOS (e) in EPCs induced by high glucose. ^*∗∗∗*^*P* < 0.001, ^*∗∗*^*P* < 0.01, and ^*∗*^*P* < 0.05 versus Con; ^##^*P* < 0.01, ^#^*P* < 0.05 versus HG. Values are expressed as the mean ± standard deviation ((a), (b), (c): *n* = 7 per group; (d), (e): *n* = 3-4 per group). Scale bar = 100 *μ*m. Con, control; HG, high glucose; Aca, acarbose.

To investigate whether high glucose was a direct factor to induce EPC dysfunction in diabetic mice and acarbose was able to prevent this change, high glucose was used to induce EPC injury in vitro. It was found that the capacity of tube formation of BM-EPCs was impaired by high glucose. Acarbose (1 *μ*M) treatment obviously improved the impaired EPC function (0.82 ± 0.12 versus 0.62 ± 0.04, *P* < 0.01; Figure 6(a)). As shown in Figures 6(b) and 6(c) high glucose caused decreased NO and increased O_2_^−^ levels when compared with the control, which was reversed by acarbose treatment (NO: 0.88 ± 0.14 versus 0.70 ± 0.16, *P* < 0.05, Figure 6(b); O_2_^−^: 1.17 ± 0.14 versus 1.38 ± 0.28, *P* < 0.05, Figure 6(c)). In addition, high glucose reduced p-Akt/Akt and p-eNOS/eNOS ratio, and acarbose treatment prevented these changes (p-Akt/Akt: 0.93 ± 0.19 versus 0.74 ± 0.06, *P* < 0.05, Figure 6(d); p-eNOS/eNOS: 0.84 ± 0.12 versus 0.70 ± 0.09, *P* < 0.05, Figure 6(e)).

### 3.6. MK-2206 Prevented the Role of Acarbose in EPCs In Vitro

**Figure 7:**
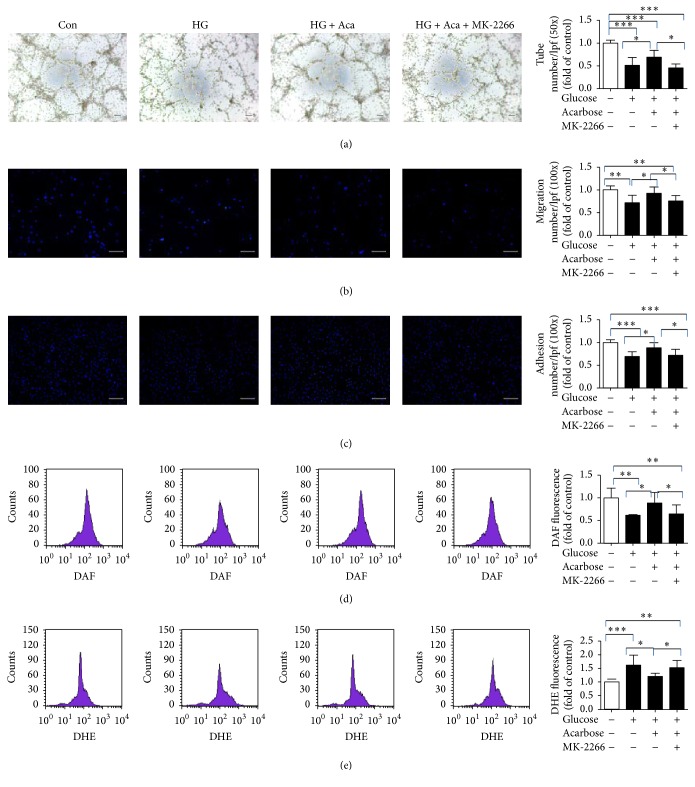
Acarbose ameliorated EPC function and suppressed intracellular ROS levels via an Akt dependent pathway in vitro. Acarbose (1 *μ*M) and p-Akt inhibitor and MK-2206 (1 *μ*M) were added to the high glucose medium for 24 h. Measurement of tube formation (a), migration (b), and adhesion (c) capacity of BM-EPCs. Determination of intracellular NO level (d) and O_2_^−^ level (e). ^*∗∗∗*^*P* < 0.001, ^*∗∗*^*P* < 0.01, and ^*∗*^*P* < 0.05. Values are expressed as the mean ± standard deviation ((a), (b), (c): *n* = 6 per group; (d), (e): *n* = 7 per group). Scale bar = 100 *μ*m. Con, control; HG, high glucose; Aca, acarbose; MK, MK-2206.

To get insight into the possible mechanism of acarbose mediated effects in EPCs under high glucose in vitro, MK-2206, a p-Akt inhibitor [30], was used. It was found that MK-2206 abolished the enhanced EPC function mediated by acarbose (tube formation capacity: 0.46 ± 0.08 versus 0.69 ± 0.15, *P* < 0.05, Figure 7(a); migration: 0.76 ± 0.12 versus 0.92 ± 0.14, *P* < 0.05, Figure 7(b); adhesion: 0.72 ± 0.13 versus 0.88 ± 0.11, *P* < 0.05, Figure 7(c)). Besides, MK-2206 pretreatment prevented the changes of NO and O_2_^−^ produced by acarbose (NO: 0.65 ± 0.20 versus 0.89 ± 0.23, *P* < 0.05, Figure 7(d); O_2_^−^: 1.52 ± 0.27 versus 1.21 ± 0.12, *P* < 0.05, Figure 7(e)). These findings suggested that the beneficial role of acarbose was possibly through Akt/eNOS signaling pathway.

## 4. Discussion

The major findings we showed in the present study are that (1) acarbose accelerated wound healing and stimulated angiogenesis in T2DM, accompanied by improved BM-EPC functions (including tube formation, migration, and adhesion) and increased NO production and decreased O_2_^−^ production in BM-EPCs; (2) in vitro, acarbose improved high glucose-mediated EPC dysfunction and enhanced intracellular NO level and impeded increased O_2_^−^; and (3) the beneficial effects of acarbose were mediated at least in part through activation of Akt/eNOS signaling.

Wound healing is a complex pathophysiologic process responding to tissue injury, which involves a cascade of interaction of various cell types, growth factors, cytokines, and other molecules [31]. Individuals with DM usually have delayed wound healing and vascular insufficiency in relation to endocrine disorder [3]. Additionally, chronic diabetic individuals are susceptible to developing refractory foot ulcer, a significant public-health problem responsible for lower-extremity amputation [32]. The process of angiogenesis plays a critical role in restoration of tissue integrity, during which the precursors of endothelial cells, especially EPCs, are involved to form new blood vessels [25, 31]. However, mechanisms of the angiogenesis remain an important obstacle yet to be elucidated.

Acarbose, as AGI, is one of the safest antidiabetic agents available, which is commonly prescribed for treatment or prevention of T2DM [33]. It is found that acarbose can serve as a protector to reduce endothelial impairment caused by hyperglycemia and thus help to improve cardiovascular outcomes [34]. Other hypoglycemic agents, such as biguanides, dipeptidyl peptidase 4 (DPP-4) inhibitors, and thiazolidinediones (TZDs), have also been demonstrated to improve diabetes-related endothelial dysfunction [17, 22, 35]. In this study, we found that pharmacological administration of acarbose contributed to significant improvement of diabetes-related impaired wound healing and reduction of EPC impairment in diabetic* db/db* mice.

As aforementioned, EPCs, critical drivers of endothelial homeostasis and regeneration, can home to ischemic tissue and play an essential role in vasculogenesis and vascular homeostasis [36–38]. Importantly, both local and systematic administration of EPCs could significantly improve angiogenesis and wound healing [25]. These evidences suggest that improved EPC function and increased circulating EPC number may play critical roles in individuals with diabetes-related vascular complications during antidiabetic therapy with certain hypoglycemic agents. We observed, in our work, that acarbose treatment induced significant increment of circulating EPCs and significant improvement of cell functions (tube formation) in both* db/db* mice-derived EPCs and in vitro high glucose stimulated EPCs. These results suggest that the reduced EPC impairment and subsequent increment of local angiogenesis in wounded area, as observed in this study, may at least in part be attributed to the accelerated wound healing in diabetic* db/db* mice produced by acarbose treatment.

It has been shown that eNOS critically regulates EPC function, and reduction of intracellular NO and increased intracellular O_2_^−^ may represent a major mechanism underlying EPC dysfunction [29, 39–41]. Moreover, the loss of eNOS-derived NO production is recognized as the main cause of EPC impairment [38]. Previous studies have reported that eNOS, the serine/threonine kinase Akt, is an upstream effector of eNOS activation in endothelial cells [42–45]. Interestingly, it has been documented that diabetic rats fed with acarbose showed improved Akt activation in adipocytes provoked by insulin [46], and similar phenomenon was also observed in cardiac tissue of acarbose-treated obese rats [47]. Thus, it is tempting to speculate that Akt/eNOS signaling pathway may be possibly involved in the protective effects of acarbose on EPCs. We observed, in the present study, that acarbose treatment induced significant increased activation of eNOS and intracellular NO levels and consequent reduction in intracellular O_2_^−^ levels. Moreover, phosphorylated Akt was significantly inhibited in BM-EPCs derived from* db/db* mice compared with the control group. Correspondingly, a significant difference in the level of p-eNOS was observed between the* db/db* group and the control group. In vitro, high glucose induced significant EPCs dysfunction accompanied by decreased intracellular NO and increased O_2_^−^ levels. However, acarbose treatment prevented the above changes, and Akt inhibition by MK-2206 diminished the protective role of acarbose in high glucose-induced EPC impairment. These results suggested that acarbose may protect diabetes-related EPC impairment by activating the Akt/eNOS signaling pathway.

In summary, our results demonstrated that wound healing, angiogenesis, and EPC function were impaired in* db/db* mice. Acarbose treatment could reverse the above pathological changes, which was possibly related to Akt/eNOS pathway. Further studies are required to better understand the mechanisms of this beneficial effect produced by acarbose administration.

## Supplementary Material

A complete methodological description of identification of the mouse leptin receptor mutation by PCR analysis.

## Abbreviations

DM: Diabetes mellitus
T2DM: Type 2 diabetes mellitus
EPCs: Endothelial precursor cells
BM-EPCs: Bone marrow-endothelial precursor cells
SDF-1*α*: Stromal derived factor-1 alpha
NO: Nitric oxide
O_2_^−^: Superoxide
eNOS: Endothelial nitric oxide synthase
AGI: *α*-Glucosidase inhibitor.
