# Supplementary material for: Acarbose Accelerates Wound Healing via Akt/eNOS Signaling in db/db Mice
Source: Oxid Med Cell Longev. 2017 Mar 8;2017:7809581. doi: 10.1155/2017/7809581 (PMC5360971; doi:10.1155/2017/7809581)
Supplement: Supplementary file 1 — A complete methodological description of identification of the mouse leptin receptor mutation by PCR analysis. [file 7809581.f1.pdf]

SUPPLEMENTAL MATERIAL

METHODS

Identification of the mouse leptin receptor mutation

The tails of mice were used for genomic DNA extraction by standard SDS/proteinase K lysis and the phenol/chloroform extraction method [1]. Extracted genomic DNA was used as a template for PCR amplification. The PCR samples comprised 50 ng genomic DNA, 0.12 µL rTaq (Takara, Japan) plus 10× PCR buffer, 1 µL $MgCl_2$ (25 mM), 0.25 µL dNTPs (10 mM), 1.2 µL BS (5M), 2.63 µL dd H_2O and 0.3 µL primers (25 µM). Target DNA was amplified in a Cyclogene Dri-Block® cycler (Techne Cambridge Ltd., U.K.), with initial denaturation at 94 °C for 3 min followed by 35 cycles of 94 °C for 30 sec, 61°C 30 sec, and 72 °C for 30 sec and a final elongation period at 72 °C for 2 min. The PCR products were analyzed by electrophoresed on a 3% agarose gel.

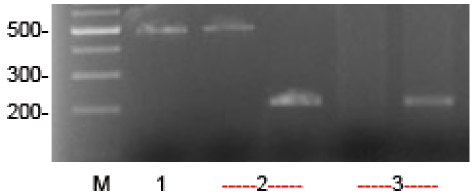

Supplementary FIGURE 1: Detection of leptin receptor mutation by agarose gel electrophoresis. Three genotypes (+/+, *db*/+, *db*/*db*) were tested. The 481 bp PCR products were amplified from normal (+/+) mice; while the 200 bp PCR products were amplified from homozygote (*db*/*db*) mice; and the 481 and 200 bp PCR products were amplified from heterozygote (*db*/+) mice. M: 100 bp ladder; 1: C57BL/KsJ; 2: BKS.Cg-m/+ $Lepr^{db}$/J *db*/+ mice; 3: BKS.Cg-m +/+ $Lepr^{db}$/J *db*/*db*.

Supplementary TABLE 1: PCR primer sequences.

Primer Sequences (5'→3')	Primer Type
GCTGCAGAATGGACGGTTGA	Common
GCAGTGCACAGGCTCAGGAA	Wild Reverse
AGCCACTACAATCCACCCCTTG	Mutant Reverse

References

[1]. SH Oh, H Nam, JG Suh. A high resolution genetic mapping of the faded (fe) gene to a region between D10mit156 and D10mit193on mouse chromosome 10. *Lab Anim Res*. 2013; 29(1): 33-38.
